# Understanding the mechanics and balance control of the carrying pole through modeling and simulation

**DOI:** 10.1371/journal.pone.0218072

**Published:** 2019-06-07

**Authors:** Tong Li, Qingguo Li, Tao Liu

**Affiliations:** 1 School of Mechanical Engineering, Zhejiang University, Hangzhou, China; 2 Department of Mechanical and Materials Engineering, Queen’s University, Kingston, ON, Canada; Istituto Italiano di Tecnologia Center for Micro BioRobotics, ITALY

## Abstract

The carrying pole has existed as a load carrying tool for thousands of years and is still popular in many parts of Asia. Previous studies attempted to determine whether the elasticity of the carrying pole is energetically beneficial compared with other load carrying methods. However, conflicting results indicate that the effects of the carrying pole stiffness on the carrier are still unclear. The carrying pole exhibits more complex characteristics beyond stiffness, which invites further investigation. As the first step towards the goal, this paper explores the underlying mechanics of the carrying pole, including the structural and dynamic properties, to determine its impact on the carrier. The structure of the carrying pole is modeled and characterized by pole length, pole stiffness, and length of suspension rope. We argue that maintaining the pole’s balance should be a major prerequisite during load carriage and that active feedback control from the carrier is required. Simulations reveal that the structural parameters of the pole have significant influences on the pole’s balance and the interaction between the pole and the carrier. This work suggests mechanical characteristics of the carrying pole can potentially have an extensive impact on gait mechanics and energetics of the carrier.

## Introduction

Transportation plays an important role in the development of human society as it enables trade between people at different locations. Advancement in vehicles has largely benefited the transportation system by increasing its speed and efficiency. However, manual load carriage is still an essential way of moving things around in our society. In some cases, manual load carriage may be the only available option for transporting goods, for example in mountain areas and congested markets where machines and vehicles cannot reach. Humans carry load using their head [1], hands [2], feet [3], etc. or utilize tools including vests [4], poles [5], packs [6], etc. Among different manual load carrying methods, the carrying pole is very commonly used in East Asian countries such as China and Vietnam from ancient times until today [7]. Archaeological evidence shows that carrying pole usage dates back to Ancient Egypt times (2300BC) [8, 9] and the Han Dynasty in China (206BC-220AD) [10]. Its prevalence starts to draw attention from academia in recent years aiming to discover the advantages and disadvantages of carrying poles in contrast to other load carrying methods [11, 12].

Previous experimental studies have investigated human energetic performance using carrying poles. Bedale [12] was perhaps the first that compared the energetics between carrying loads with poles and other methods (including carrying loads on the hip, in trays, on the head, etc.). This study found that carrying loads with a pole (The “pole” was termed as “yoke” in some literature, but we use “pole” for consistency in our paper) on the shoulders costs the least amount of metabolic energy, compared to other load carrying methods. Datta et al. [2] compared the energetic cost of carrying an identical weight in seven different modes (Head, Rucksack, Double Pack, Rice Bag, Sherpa, Pole, and Hands) and found using poles ranked second to last, where using hands was last place. They mentioned that the bamboo pole caused significant discomfort, which might account for the inferior performance. Balogun et al. [11, 13] also looked into the cardiorespiratory response and metabolic efficiency of carrying loads with the headpack and two types of poles (the transverse pole and the frontal pole). They found that the head pack and transverse pole performed similarly and that both were more economical than the frontal pole. This study was perhaps the first to recognize that the elastic property of the pole should be further investigated. Kram [5] studied the performance of running with compliant poles and found no energetic advantage over backpacks, but a significant reduction of peak vertical interaction force on the shoulder. A more recent study found that a compliant pole costs less energy (5.0%) than a stiff steel pole during walking [14].

Previous experiments have presented inconsistent or even contradictory results on the performance of carrying poles, when compared with other load carrying methods. However, many factors of these studies were controlled differently, such as subject’s skills, types of locomotion (walking or running), and mechanical properties of the poles. It is also unclear whether these factors account for these inconsistent observations. Nevertheless, the inconsistency indicates that the performance of carrying poles might be prone to many factors and the results in those studies should be compared and interpreted with caution. Further investigation is still necessary to discover the underlying mechanisms related to the performance of the carrying pole.

Recent studies have suggested that elasticity is an important characteristic of a carrying pole and that it can reduce the energetic cost [14] and peak interaction forces at the shoulder [15]. The elasticity of a pole is often conceptualized as springs suspending the load in the vertical direction, according to classic beam theory [15, 16]. In this case, the carrying pole is believed to operate similarly to the elastic backpack, where carried load is also elastically suspended in the vertical direction as well [17]. In the case where the load is suspended by a rubber band in a backpack and oscillates out of phase with the body, it has shown to reduce the metabolic cost by 6.2%, compared to the fixed condition [17]. Modeling studies have also verified that a low-stiffness springy connection between the load and body can reduce the energetic cost of load carriage [18–20]. Model-based analysis has also provided some guidelines for effective bamboo pole design for reducing peak shoulder forces [15].

However, these recent studies on pole elasticity show conflicting results in some aspects. For example, Schroeder et al. [16] measured the stiffness of traditional bamboo poles used in Vietnam and found that none of these poles (14 in total) fall within the recommended parameters for reducing peak shoulder forces according to [15]. Carrying loads with a compliant bamboo pole costs less energy than using a steel pole, but still costs more energy than conventional fixed-load carrying methods [14]. The authors argued that this was because the subjects were unskilled using poles for carrying weight [14]. Nevertheless, it is unclear what factors make the compliant pole more difficult to acclimatize to, compared to the elastic backpack, which costs less energy than a traditional rigid backpack [17]. It is noted that subjects carrying the elastic backpack adopted the same preferred walking frequency as carrying a fixed-load backpack [17, 21], while the preferred walking frequency using a compliant pole was significantly higher than using a stiff pole [14]. Moreover, carrying the stiff steel pole resulted in a higher walking frequency than the no-load walking condition [14], whereas the traditional backpack usage had little influence on walking frequency [22, 23].

To understand the profound pole-carrier interaction it requires a full understanding of the mechanics of both the carrying pole and the carrier. The contradictions in previous experiments imply that the stiffness of the pole cannot solely clarify the influence of a carrying a pole on a carrier and that other important factors of the carrying pole are likely to influence the carrier. However, little attention has been given to pole attributes beyond pole stiffness. Some potential factors include carrying styles, pole length, and pole balance. In order to broaden our understanding of the carrying pole, this paper looks into the mechanics of pole load carriage from a new perspective focusing on pole structure and dynamics. We also argue that controlling the balance of the carrying pole may be a critical factor, which affects gait mechanics and energetics of the carrier during dynamic pole-carrier interaction. In this paper, modeling and simulations are performed to explore the influence of pole structure on pole balance and pole-carrier interaction. These new perspectives may facilitate a better understanding of the underlying mechanisms of the carrying pole and draw further attention in social science and engineering to study this historic carrying tool.

## Overview of the human-pole-load system

**Fig 1:**
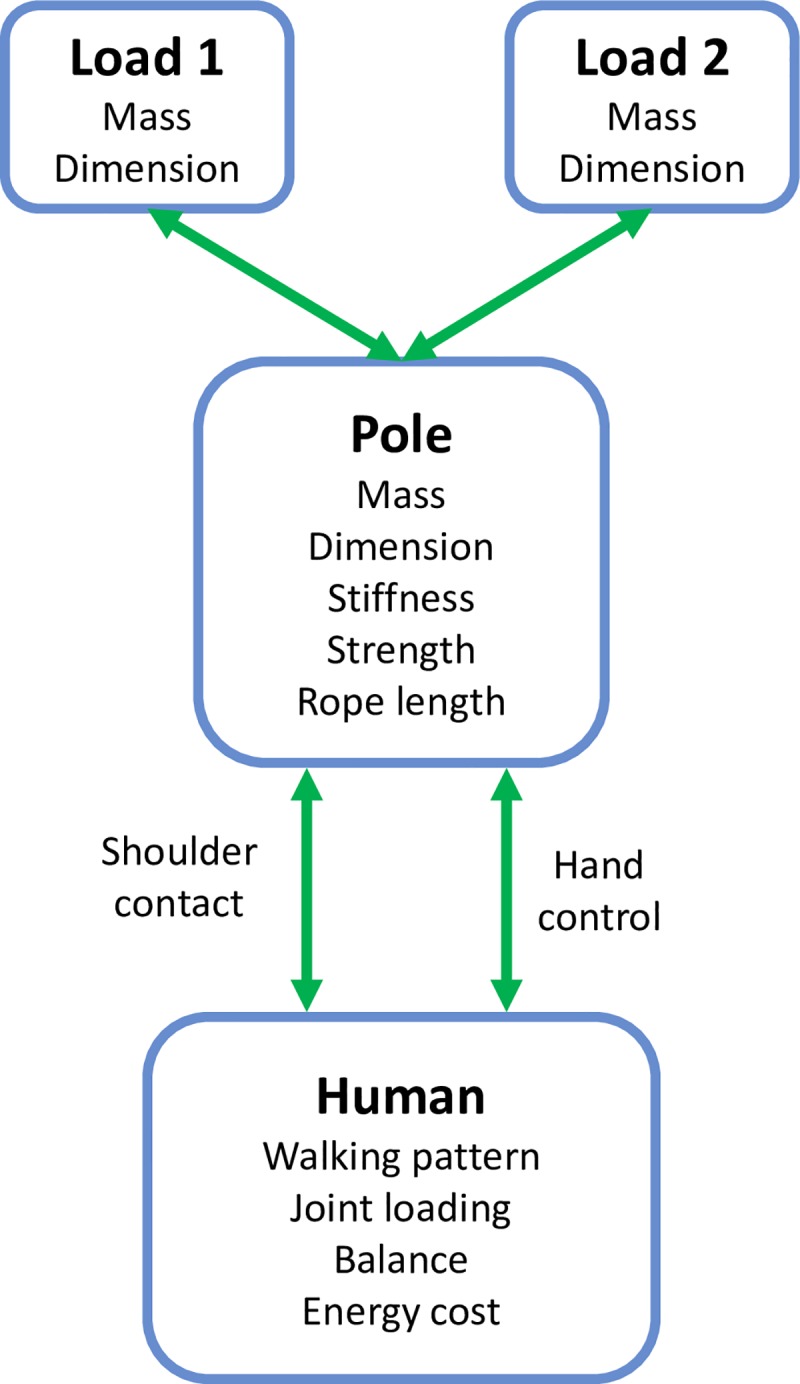
An abstract diagram of the human-pole-load system. Two loads are attached to the pole and the pole interfaces with the human at the shoulder and hands. Some attributes of each component are also listed.

Carrying loads with a pole forms a human-pole-load system as depicted in Fig 1. The pole is usually used to carry heavy (in mass) and bulky (in dimension) loads such as water, crops, and materials for construction. From the carrier’s perspective, interaction with the pole will influence their walking pattern, joint loading, balance, and energetic cost. These considerations most likely lead to requirements for pole design to achieve better performance during load carriage in aspects such as comfort, convenience, safety, and efficiency.

We believe the primary reason for people choosing this tool, or inventing this tool, is related to its structure. The most distinct advantage of using a pole, compared with backpacks or some other tool, lies in the balanced configuration: where two loads are balanced at each end of the pole. Therefore, the resultant force vector (downward) is near the body’s center of mass and lumbar joint. This means the pole will impose only a small moment on the lumbar joint and reduced effort is required to balance the upper trunk. In backpack load carriage, the trunk will lean forward to keep the combined center of mass of backpack and trunk over the pelvis, which results in higher trunk muscle activity and higher spine load [6]. Injuries such as low-back pain are also more likely to occur in this scenario. In addition, the load carried by a carrying pole can be over 100% body weight. Assuming the same load is carried with a backpack, it may not be possible to balance such a load using postural changes with the trunk. It may also require an unbearable spine joint moment. In this way, the pole can increase the carrying capacity significantly. The double pack which consists of two packs distributed in the front and back of the carrier [24] can also provide a similar balanced configuration but may suffer from problems like ventilation [25] and ease of donning and doffing the load.

Another advantage of the pole structure is its ability to carry bulky loads, which is required in agriculture and other fields. For example, transporting crops demands a large amount of space. Using methods such as hands or packs will severely impact the task’s efficiency. Even though the pole’s length may result in some sacrifice in maneuverability, its pros outweigh the cons in a case when large loads are needed to be carried.

When comparing carrying loads using hands, the advantage of using a pole is that it requires little arm force, as the load is supported primarily by the trunk like a backpack. Large muscles groups in the trunk do not get fatigued as easily as smaller muscles groups in the arm [25]. The control force from hands is still needed, but the force amplitude is much smaller compared with directly holding the loads with hands.

The ropes used to suspend the load are also beneficial as they allow the load to be at a low position. In this case, the carrier can easily lift the load requiring only a small ground clearance. The length of ropes can be adjusted to adapt to different terrain. The loads can also be easily put down on the ground. Other tools such as the backpack will be more challenging to pick up and put down.

Some trade-offs may arise in the selection of geometric parameters. For example, the pole should not be too short when carrying large loads [15]. The width and thickness will also affect the strength of the pole. They should not be too small when bearing heavy loads, but they should also not be too massive as this will increase the weight of the pole. An interesting phenomenon is that many poles are made from bamboo, which has a hollow structure. According to mechanical theories, the hollow structure tends to have larger strength-to-mass ratio [26]. Using hollow bamboo, properties such as lightweight and high strength can be achieved simultaneously, which is desirable for the carrier.

The advantages gained from the structure of carrying poles, as described above, are rather apparent and do not require much further mechanical analysis. It perhaps explains why the pole has existed since the time when people had little to no knowledge about mechanics and dynamics. However, these beneficial structural features also introduce some other issues and characteristics, especially when considering the dynamic condition. One particular issue that the carrier has to manage is keeping the pole in balance during body movements, particularly on rough terrain. The rope also introduces swingy motion, which may introduce challenges for maintaining stability while walking. A springy pole will also introduce vibrations during walking. Most existing studies on the mechanics of carrying poles focused on the stiffness of the pole and only considered the vertical interaction [14, 15], overlooking other important dynamic features. These features inevitably have large impacts on the carrier in various aspects such as walking pattern and energetic cost. In order to gain a better understanding of the carrying pole, we propose a pole-carriage model to analyze its dynamics. This work can serve as a foundation for further exploration on the influence of pole carriage on the user.

## Structure and modeling of the carrying pole

**Fig 2:**
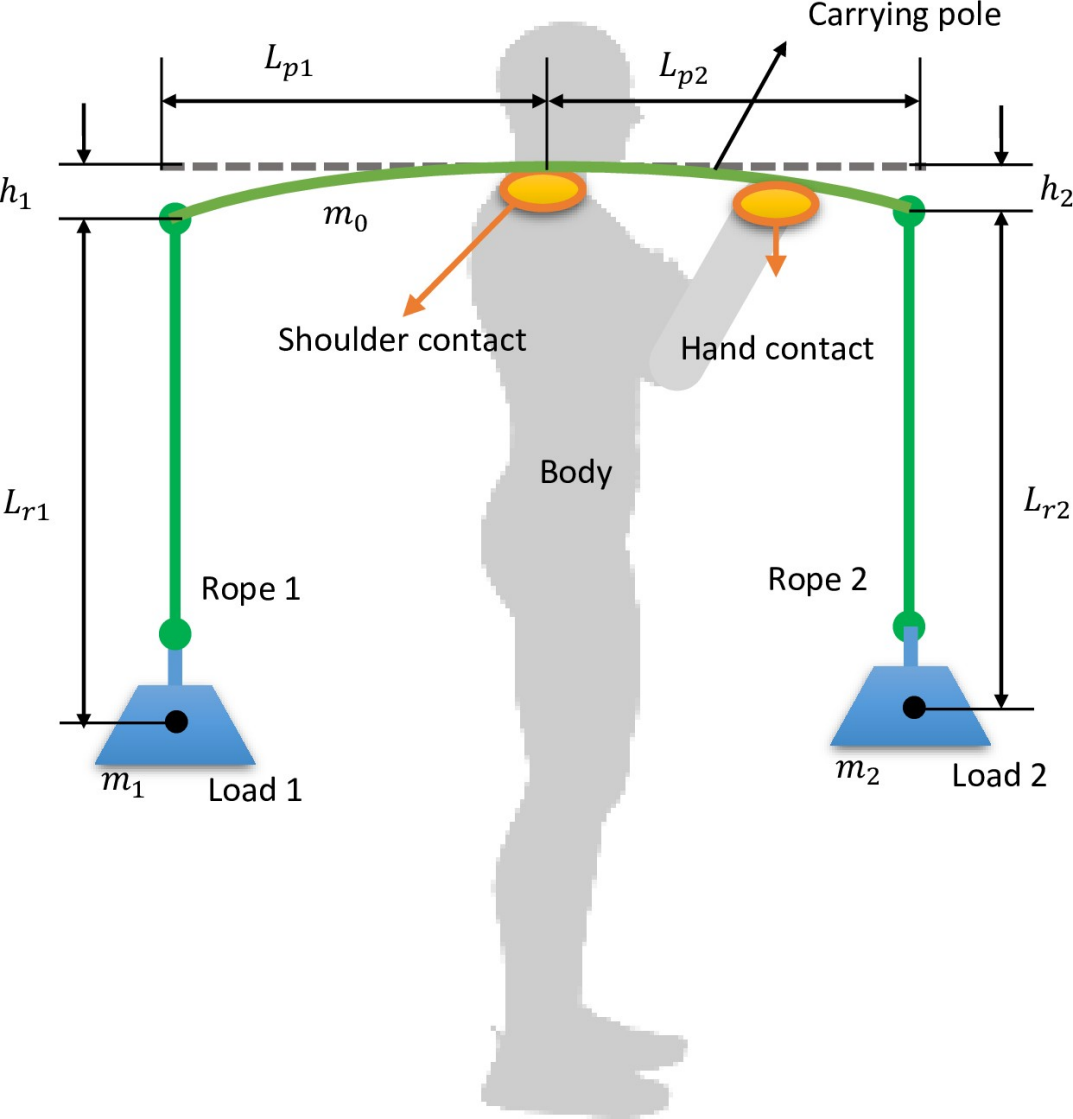
A typical pole carriage diagram. The pole is carried in the frontal style and the right hand controls the pole balance. Two loads are suspended with ropes at each end of the pole. The pole deforms due to the load weights.

In this work, we take the “frontal pole” [11] as a typical pole-carrying style for analysis (Fig 2). Some other carrying styles such as transverse pole are also used in some cases and can be explored in further work. Since human walking is usually studied in the sagittal plane, this configuration helps to simplify the overall model as the pitch motion of the pole is also in the sagittal plane. The motion of carried loads in the medial-lateral direction is thus neglected in this work. In the human-pole-load system, two loads of similar mass (*m*_1_,*m*_2_) are suspended at the two ends of the pole via ropes at the distances of *L*_*p*1_ and *L*_*p*2_ to the shoulder. The distances from the attachment point of the rope to the load’s center of mass can be regarded as the effective pendulum length (*L*_*r*1_,*L*_*r*2_). Due to the weight of the loads, the pole will deform like a beam resulting in a vertical displacement (*h*_1_,*h*_2_) for each end (horizontal displacement is negligible). According to the beam theory [26], the static deflection at each end (vertical displacement) due to the load can be calculated as:

$$h_{1}=\frac{m_{1}gL_{p1}^{3}}{3EI},h_{2}=\frac{m_{2}gL_{p2}^{3}}{3EI}$$ (1)

assuming the pole is an ideal cantilever beam where *E* is Young’s modulus and *I* is the second moment of the area [15]. In this case, we can derive the stiffness of the pole at each side:

$$k_{1}=\frac{3EI}{L_{p1}^{3}},k_{2}=\frac{3EI}{L_{p2}^{3}}$$ (2)

We extract three major parameters to characterize the dynamics of the carrying pole.

- Length of the pole (*L*_*p*1_,*L*_*p*2_)
- Stiffness of the pole (*k*_1_,*k*_2_)
- Length of the rope (*L*_*r*1_,*L*_*r*2_)

Note that we are not including the parameters of the load or the carrier and focusing only on parameters related to the pole itself. We also separate the stiffness and length of the pole since either can be varied individually whilst keeping the other constant, for example by altering *E* or *I*. In this way, we can explore the influence of stiffness and length independently.

**Fig 3:**
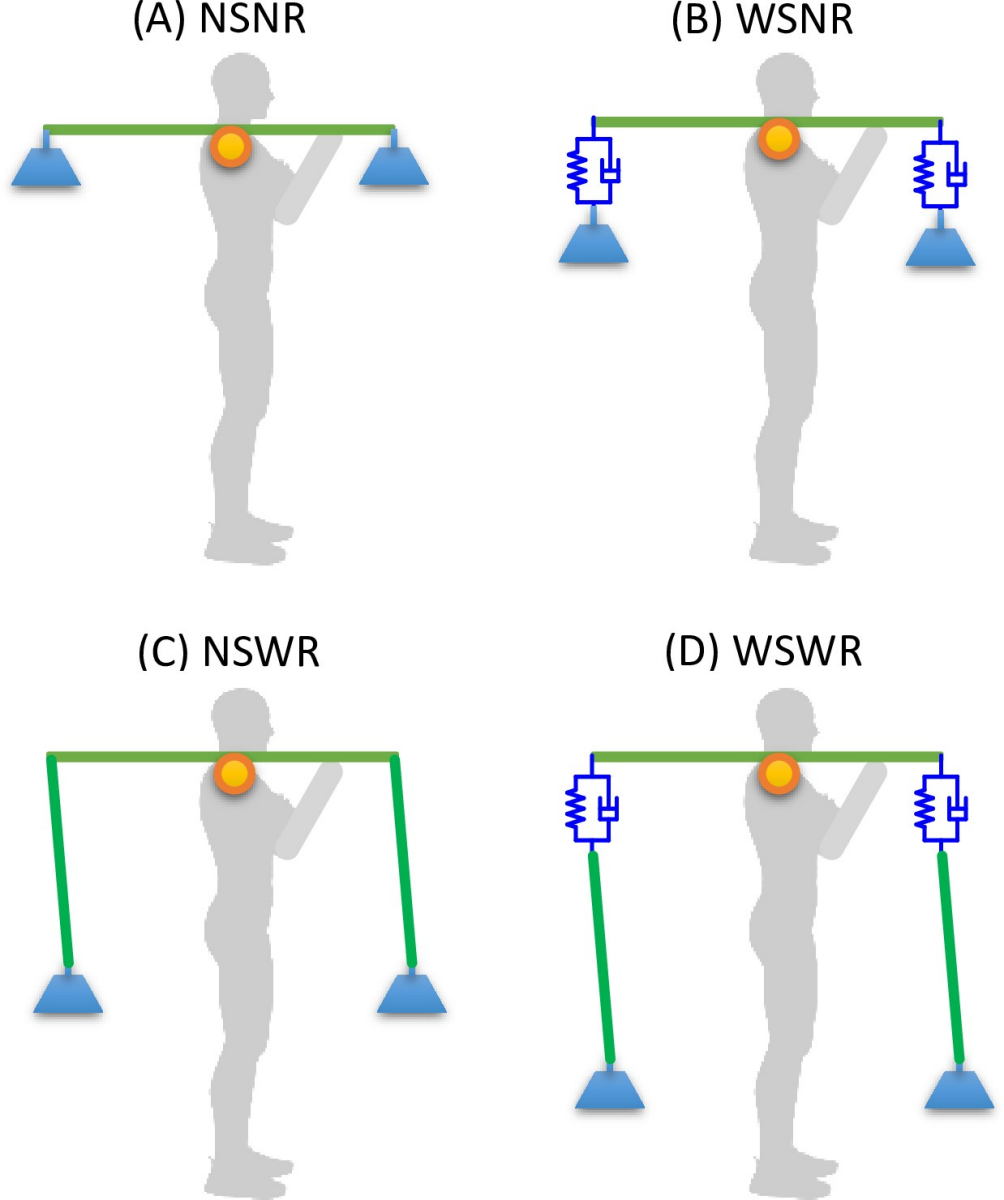
Four abstract types of the carrying pole. (A) NSNR (no spring, no rope); (B) WSNR (with spring, no rope); (C) NSWR (no spring, with rope); (D) WSWR (with spring, with rope).

Based on these three parameters we have four abstract types of a carrying pole, as shown in Fig 3, assuming an ideal infinitely large stiffness or a rope of zero length. Note that we always assume a finite and non-zero length of the pole. Also, since the pole deflection mainly exists in the vertical direction, according to beam theory, we use a vertical spring-damping unit to model the bending stiffness of the pole. The four types of carrying pole are:

1. NSNR (no spring, no rope): the pole is ideally rigid and with no rope (Fig 3A);
2. WSNR (with spring, no rope): the pole is elastic and with no rope (Fig 3B);
3. NSWR (no spring, with rope): the pole is ideally rigid and with rope (Fig 3C);
4. WSWR (with spring, with rope): the pole is elastic and with rope (Fig 3D).

By comparing these four types of model we can explore the effects of each pole parameter independently. These four types apply the same force to the body in the static condition, indicating the effects of these factors are only prominent during dynamic conditions, such as walking.

**Fig 4:**
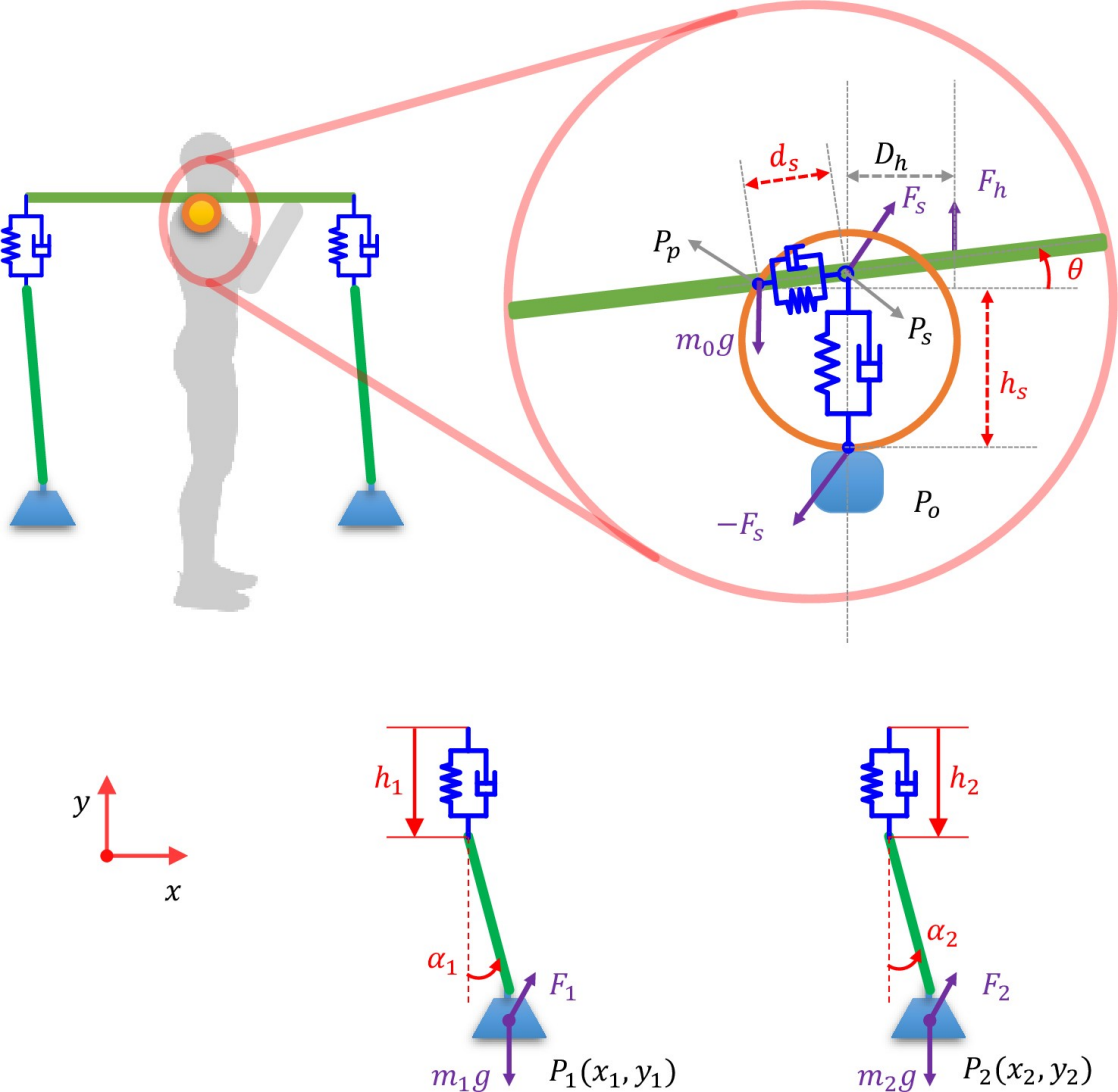
The model of the human-pole-load system. The free body diagram of the system and the diagram of the contact model that contains two spring-damping units.

The interaction between the pole and human occurs at two interfaces: the shoulder contact point and the hand contact point (Fig 1). The shoulder supports the weight of the carried mass while the hand is used to maintain pole balance. The contact region between the shoulder and pole provides some margin of error for pole balance and also allows for some inaccuracies in pole placement. However, here we assume the contact area is very small and consider it as a point contact to simplify the analysis. Ideally, the pole can be balanced somewhere near the midpoint (named balance point). However, rollover will happen if the contact point shifts away from the balance point. Thus the contact point should not be fixed at the balance point. On the other hand, interaction forces are required to transfer the body motion to the pole. Inspired by the foot contact model in [27, 28], we developed a contact model for the shoulder as shown in Fig 4. Two spring-damping units are used to describe the dynamic interaction between the shoulder and the carrying pole. One spring-damping unit operates in the vertical direction and is directly connected to the shoulder. We assume the trunk is always upright and thus it describes the vertical deformation of the shoulder due to the pole. The second spring-damping unit is along the pole direction, rather than horizontal, as the contact area of the shoulder and the pole is always tangential with respect to the pole orientation. This configuration allows for the existence of relative motion and interaction force between the shoulder and the pole. The need for hand control on the pole makes pole load carriage significantly different from other load carriage scenarios, such as using a backpack which does not require hand control to keep its balance. A vertical control force (*F*_*h*_) at a distance of *D*_*h*_ in front of the shoulder is used to simulate the hand’s control in maintaining the carrying pole’s horizontal orientation. For simplicity, we assume the active control from the human, to keep the pole in balance, comes only from the hand force. Further work may investigate the body’s (especially shoulder) ability to change the pole’s balance.

Fig 4 shows the free body diagram (FBD) of the human-pole-load system for the WSWR model. The other three simplified models in Fig 3 can be obtained by removing corresponding elements. For the WSWR model, the system general coordinates are **x** = (*h*_*s*_,*d*_*s*_,*θ*,*h*_1_,*θ*_1_,*h*_2_,*θ*_2_) and the equation of motion (EOM) of the system is:

$$M(x)\ddot{x}+C(x,\dot{x})+G(x)=B_{1}\left[\begin{array}{c} \ddot{x_{o}}\\ \ddot{y_{o}} \end{array}\right]+B_{2}[F_{h}]$$ (3)

where **M**∈*R*^7×7^ is the inertia matrix, **C**∈*R*^7×1^ has the Coriolis, centrifugal and feedback control terms, **G**∈*R*^7×1^ has the gravity terms, **B**_**1**_∈*R*^7×2^ is the Jacobian matrix for the shoulder acceleration input and **B**_**2**_∈*R*^7×1^ is the Jacobian matrix for the control force. Details on the derivation of the EOM can be found in the Appendix A.

**Fig 5:**
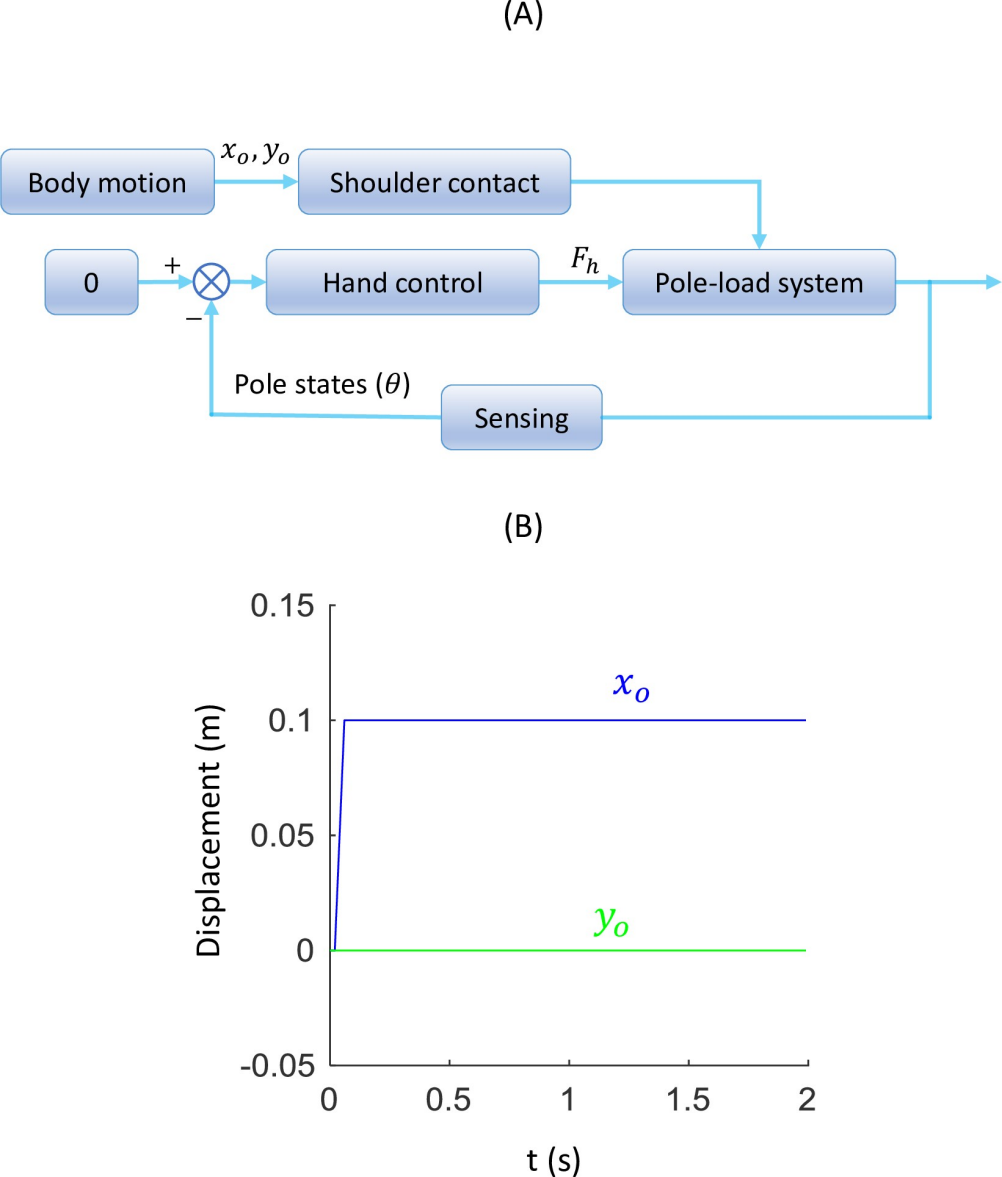
Balance control of the carrying pole. (A) The closed-loop control system depicting the pole carriage scenario. (B) The trajectory of the shoulder in horizontal (*x*_*o*_) and vertical direction (*y*_*o*_) for testing the step response of the pole model.

The human-pole-load system can be modeled as a closed-loop feedback control system (Fig 5A). The zero reference input means the carrier’s control goal is to keep the pole in the horizontal orientation. The motion of the shoulder (*P*_*o*_(*x*_*o*_,*y*_*o*_)) acts as a noise input to the pole-load system that induces pole imbalance. In reality, humans can use complex sensory systems, such as eyes, cutaneous sensors in the hands and proprioceptive sensors at the shoulder, to sense the states of the pole. Since the goal here is to simply maintain the pole’s balance rather than exploring the exact sensory system and balance strategies that humans adopt, we assume that the carrier can acquire the pole state information precisely and in real time to determine the control force at the interface. Some processes, such as neural signal transmission and muscle activation, are also neglected. Proportional-derivative (PD) control is often used to simulate the compensatory control of humans during quiet standing [29] and steering control of a car [30] or bicycle [31]. Similarly, the control force at the hand is simulated here by a PD control law and is given in the form:

$$F_{h}=-K_{p}\theta -K_{d}\dot{\theta }$$ (4)

where *K*_*p*_ and *K*_*d*_ are the feedback control gains and *θ* and $\dot{\theta }$ are the pitch angle and angular velocity of the pole which can be sensed by the carrier’s eyes.

## Simulation and analysis

In our simulation, we assume the two loads have identical point mass (*m*_1_ = *m*_2_ = 10*Kg* and *I*_1_ = *I*_2_ = 0). The pole is initially supported at the middle point which is the equilibrium point of the pole ($L_{p1}=L_{p2}=\frac{L}{2}$, where *L* is the pole length). Variables *m*_0_ and *I*_0_ is assumed to be small but should not be zero in order to avoid singularity when solving angular acceleration. We use *m*_0_ = 1*Kg* and $I_{0}=\frac{m_{0}{\left(L_{p1}+L_{p2}\right)}^{2}}{12}$. We also consider the pole stiffness and rope length at each end to be the same (*k*_1_ = *k*_2_,*L*_*r*1_ = *L*_*r*2_). The arm to control the pole’s balance is assumed to be massless. In reality, it can be compensated by the difference in load mass or a shift of the shoulder contact point. The model is driven by the two dimensional motion of the shoulder. If the body only has vertical motion (*x*_*p*_ = 0,*y*_*p*_ ≠ 0), the pole will not roll over since the supporting position will always be at the initial equilibrium point (middle point of the pole). This indicates the imbalanced condition will come from the horizontal motion of the body which will lead to a deviation of the shoulder supporting point. We first study the response of the system under a step input of the horizontal body motion (*x*_*o*_) as in Fig 5B in the following three sections to study the balance dynamics of the system.

### Parameters of the contact model

**Fig 6:**
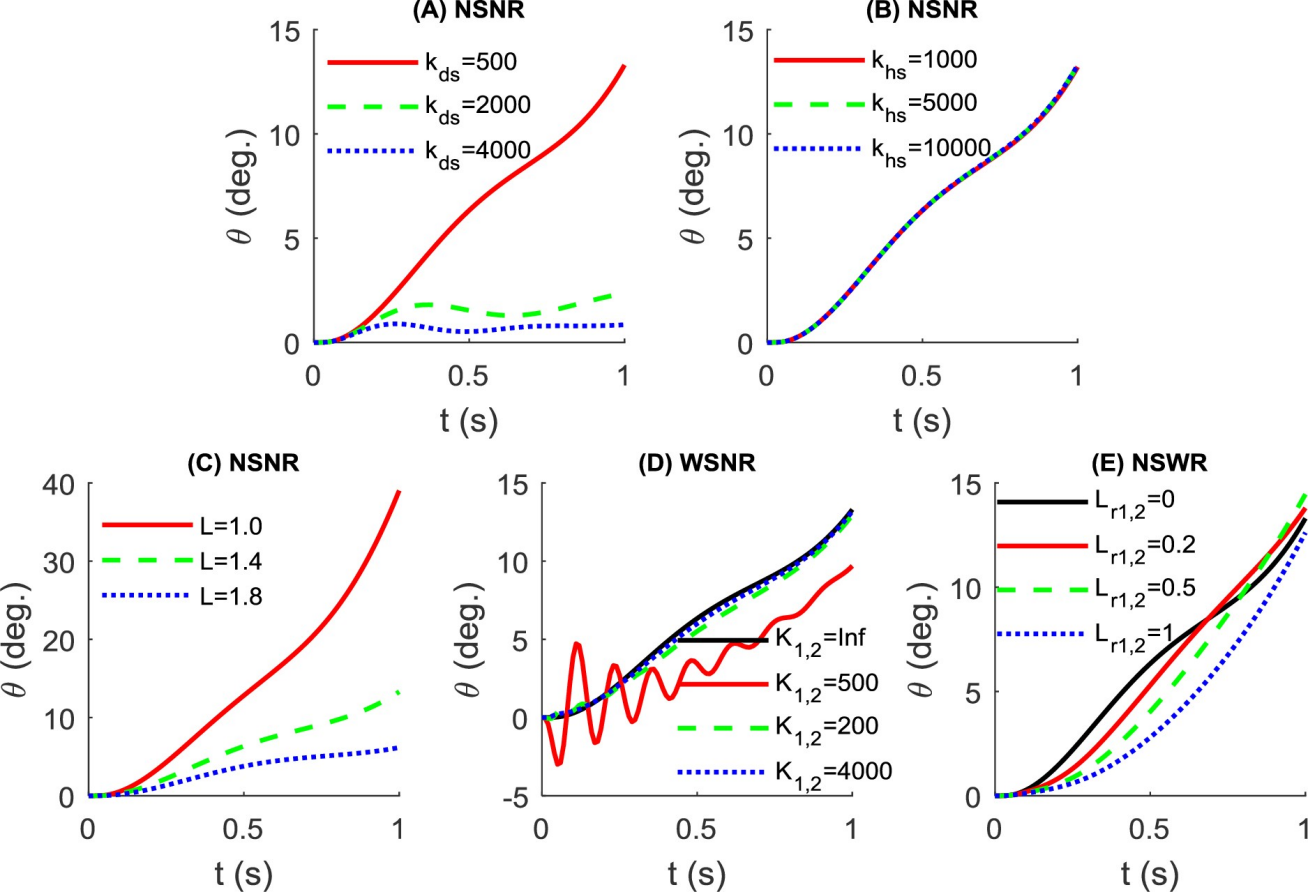
Rollover angle variation without hand control at different conditions. (A) Simulated using the NSNR model with different tangential stiffness values where *k*_*hs*_ = 10000*N*/*m*, *L* = 1.4*m*. (B) Simulated using the NSNR model with different vertical stiffness values where *k*_*ds*_ = 500*N*/*m*, *L* = 1.4*m*. (C) Simulated using the NSNR model with different pole length values where *k*_*ds*_ = 500*N*/*m*,*k*_*hs*_ = 10000*N*/*m* (D) Simulated using the WSNR model with different pole stiffness values where *k*_*ds*_ = 500*N*/*m*, *k*_*hs*_ = 10000*N*/*m*,L = 1.4*m*. (E) Simulated using the NSWR model with different rope length values where *k*_*ds*_ = 500*N*/*m*,*k*_*hs*_ = 10000*N*/*m*,L = 1.4*m*.

Intuitively, the vertical stiffness (*k*_*hs*_) would be relatively large due to the bones underneath the skin. Where as the tangential stiffness (*k*_*ds*_) may be relatively small due to the movement of soft tissues like skin. Since there is no available data on the parameters of the contact model in literature, we tested three values for tangential stiffness *k*_*ds*_ (500, 2000 and 4000 N/m, with tangential damping $c_{ds}=0.25*2*\sqrt{k_{ds}(m_{1}+m_{2})}$) and three values for vertical stiffness *k*_*hs*_ (1000, 5000 and 10000 N/m, with vertical damping $c_{hs}=0.25*2*\sqrt{k_{hs}(m_{1}+m_{2})}$) in a no hand control condition (*F*_*h*_ = 0) as shown in Fig 6A and 6B. It was found that smaller tangential stiffness values (*k*_*ds*_ = 500*N*/*m*) leads to faster rollover (around 15° in the first second). Where as the pole stays approximately at a balanced state at a larger value (*k*_*ds*_ = 4000*N*/*m*). However, the value of vertical stiffness has little to no effect on rollover. In the following investigation, we use *k*_*ds*_ = 500*N*/*m* and *k*_*hs*_ = 10000*N*/*m* when examining the influence of pole parameters on maintaining pole balance.

### Effects of pole parameters (without hand control)

Let us still assume no hand control (*F*_*h*_ = 0) and test the influence of different pole parameters on the balance of the pole. We simulated different pole length values (L = 1.0, 1.4 and 1.8m) based on the NSNR model and found that the pole will always roll over regardless of pole length (Fig 6C). However, longer poles have much slower rollover speed.

The response of the WSNR model is tested with different pole stiffness values (*k*_1_ = *k*_2_ = 500,2000 *and* 4000 *N*/*m*, $c_{1}=c_{2}=0.01*2*\sqrt{k_{1}m_{1}}$) and the results are compared with the NSNR model (*k*_1_ = *k*_2_ = *Inf*), as shown in Fig 6(D). Interestingly, lower pole stiffness (500N/m) results in slower rollover speed (less than 10 Deg. in the first second). However, there are many ripples due to the oscillations of the spring. With an increase in stiffness, the rollover speed becomes larger and similar to the rigid pole. As well, ripples in pole angle over time are reduced or diminished.

The response of the NSWR model is tested using three rope length values (*L*_*r*1_ = *L*_*r*2_ = 0.2,0.5 and 1*m*). The results are compared with the NSNR model (*L*_*r*1_ = *L*_*r*2_ = 0), as shown in Fig 6E. Generally, the rollover speeds after one second are similar at different rope lengths. However, a longer rope tends to reduce the rollover speed at the beginning (< = 0.5s).

### Effects of pole parameters (with hand control)

**Fig 7:**
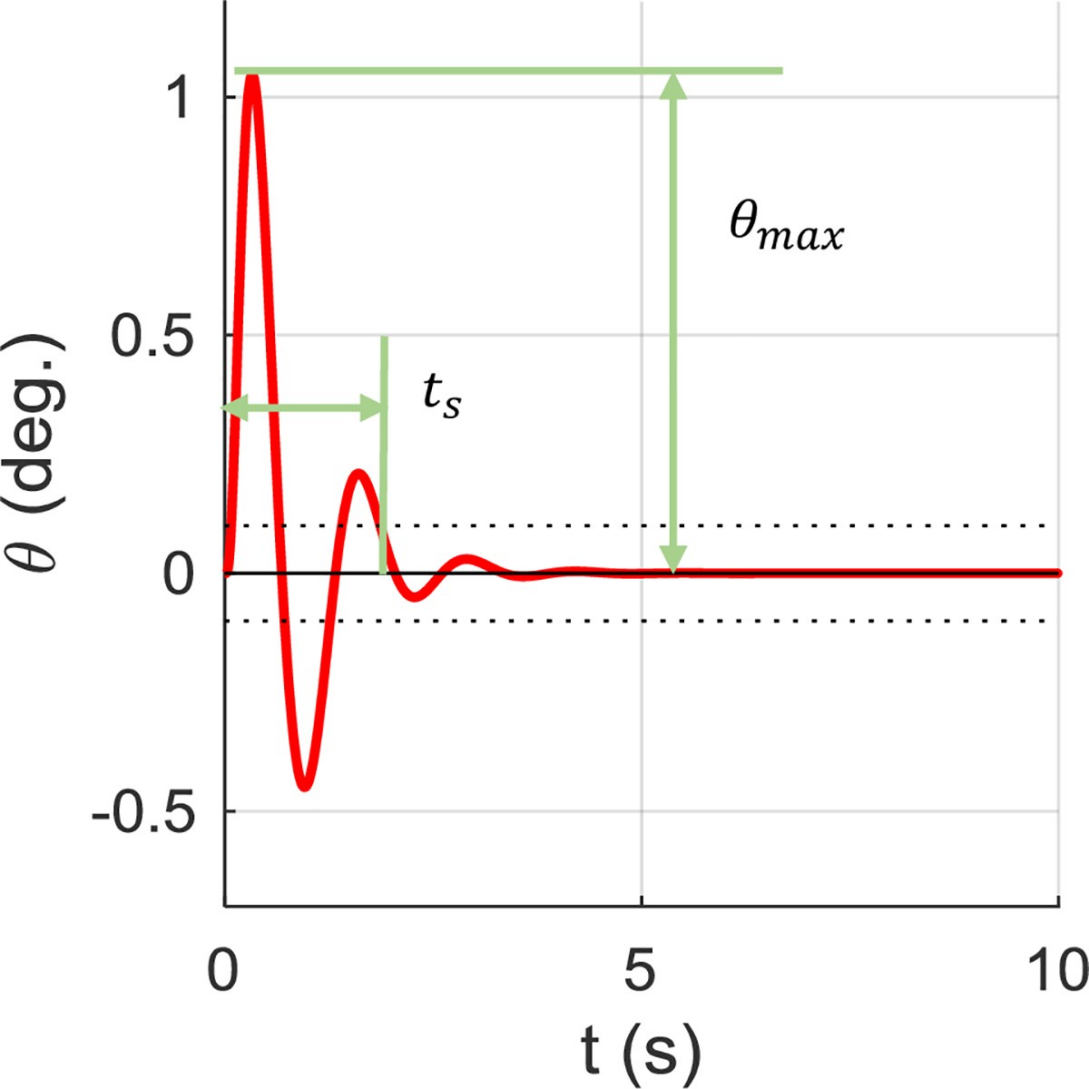
A PD controller can stabilize the NSNR model, keeping the pole angle around zero. The maximal angle *θ*_*max*_ and settling time *t*_*s*_ are also defined, as depicted, for comparison in later simulations.

We will now consider the situation with hand control. Based on the NSNR model, we implement the PD controller to prevent pole rollover under a step motion input. The performance of the controller depends on the feedback gains, which may differ for different individuals. Since our goal is to compare balance control in different pole configurations, we assume fixed gain values. We tune the feedback gains with trial and error and choose *K*_*p*_ = 1000,*K*_*d*_ = 500, which can stabilize the NSNR model and the resultant angle profile as shown in Fig 7. We also define the maximal rollover angle *θ*_*max*_ and the settling time *t*_*s*_ so that the angle enters and stays in the region of −0.1°~0.1°. We use both performance indexes when comparing different pole parameters.

**Fig 8:**
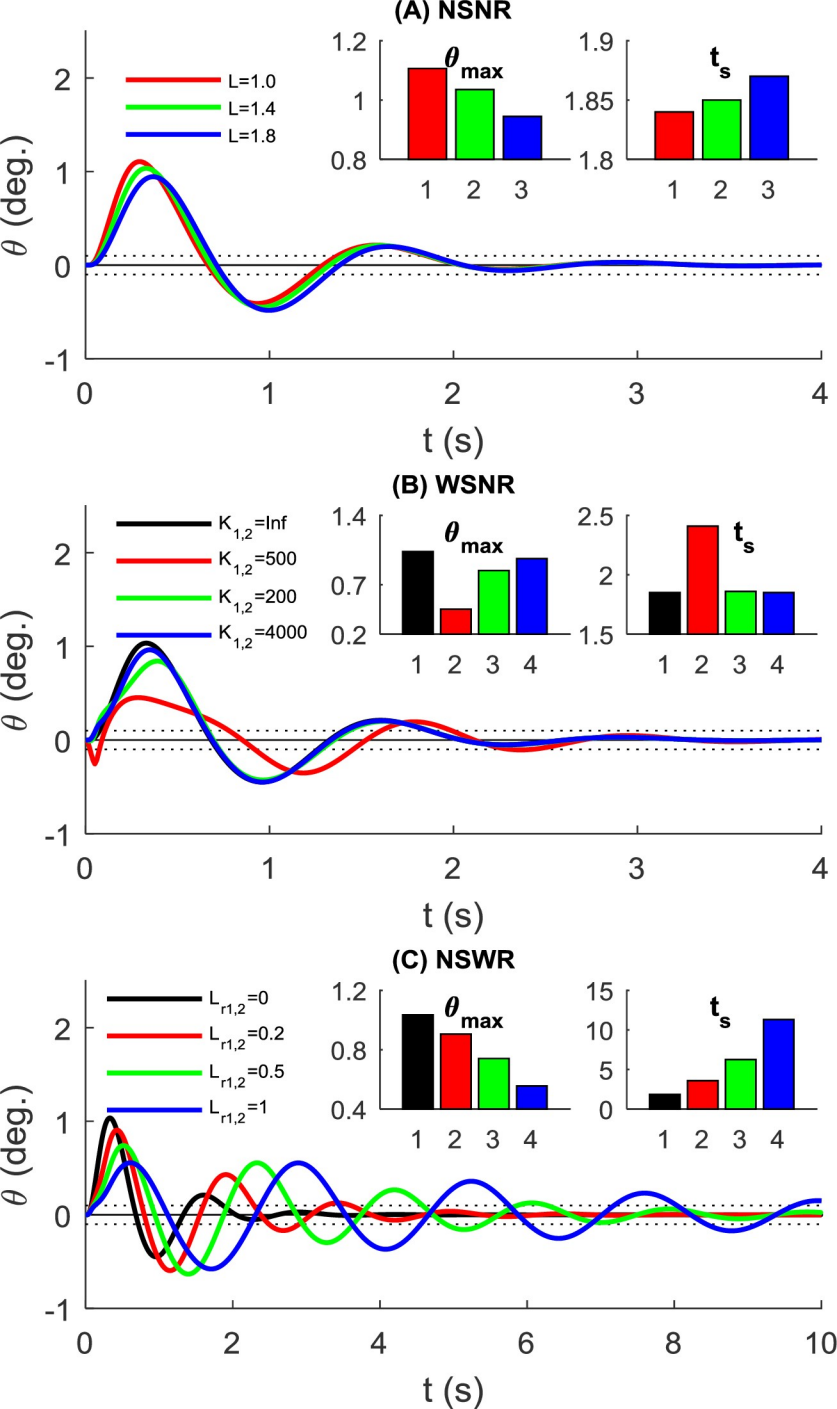
Rollover angle variation with step input and active control under different conditions. (A) Simulated using the NSNR model with different pole lengths, where *k*_*ds*_ = 500*N*/*m*, *k*_*hs*_ = 10000*N*/*m* (B) Simulated using the WSNR model with different pole stiffness, where *k*_*ds*_ = 500*N*/*m*, *k*_*hs*_ = 10000*N*/*m*,*L* = 1.4*m*. (C) Simulated using the NSWR model with different rope lengths, where *k*_*ds*_ = 500*N*/*m*, *k*_*hs*_ = 10000*N*/*m*,*L* = 1.4*m*. The max angle and settling time are also compared in bar graphs and the color of each bar corresponds to the waveform color.

With active control on the NSNR model, the pole can return to a balanced state in all three length values with a similar transient response (Fig 8A). A shorter pole shows a relatively larger maximal angle and shorter settling time.

For active control conditions, the effect of pole stiffness is shown in Fig 8B. We simulated three stiffness values. A small stiffness (k = 500N/m) tends to have a significantly smaller maximal angle but much longer settling time. Larger stiffness values (2000, 4000 N/m) perform similarly to the rigid pole, with a small reduction of maximal angle.

We tested three rope length values and found shorter ropes reduces the maximal angle but leads to a much longer settling time and significant oscillations (Fig 8C).

### Using shoulder motion during normal walking as model input

We use experimentally measured shoulder trajectory data during normal walking as input to drive the model. One subject was recruited to walk on a treadmill at the preferred frequency at a speed of 1.2m/s. Trials length was one minute. A reflective marker was attached to the right shoulder of the subject. The trajectory of the marker was captured using a VICON motion capture system. The study was approved by the Medical Ethics Committee of the School of Medicine, Zhejiang University (Project identification code: 2016–007) and written consent was obtained prior to the walking trial. The actual body motion might change more or less under different carrying conditions. However, it is hard to predict exact gait changes in response to different loading conditions. As well assuming fixed kinematics provides insights in human-machine-interaction studies [32, 33]. Thus the same shoulder trajectory profile is used as input to drive different models assuming invariant body kinematics. The results of pole angle (*θ*), control force at the hand (*F*_*h*_) and the interaction forces at the shoulder in forward (*F*_*s*−*x*_) and vertical (*F*_*s*−*y*_) directions are obtained from the simulation for comparison across different parameters.

**Fig 9:**
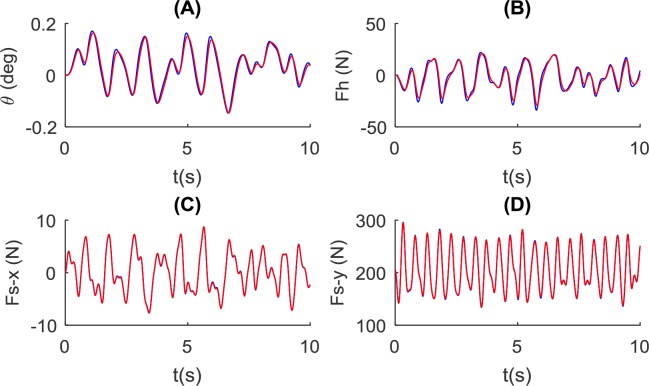
Influence of pole length on the balance of the pole and pole-carrier interaction. Simulated using the NSNR model with two pole length values (blue: *L* = 1.4*m* and red: *L* = 1.8*m*) and *k*_*ds*_ = 500*N*/*m*, *k*_*hs*_ = 10000*N*/*m*. Simulation results for: (A) the pole angle, (B) control force at the hand, and the interaction force at the shoulder in (C) forward direction and (D) vertical direction.

Two poles of different lengths (1.4m and 1.8m) are tested as shown in Fig 9. It is observed that pole length has little influence on the angle and force profiles. The longer pole shows a marginally smaller angle and hand force variation amplitude. Interaction forces in both directions are almost identical under the two pole length conditions.

**Fig 10:**
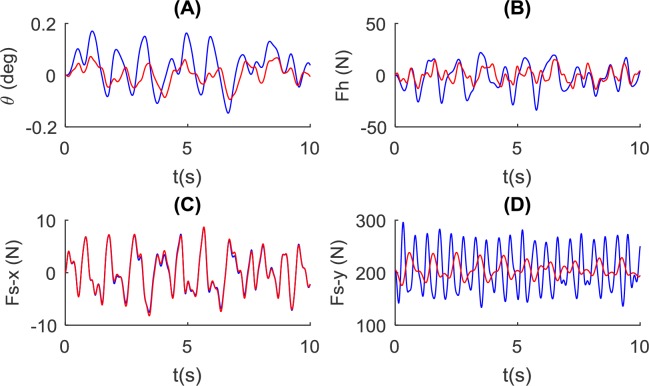
Influence of pole stiffness on the balance of the pole and pole-carrier interaction. Simulation of the rigid condition (in blue, using the NSNR model and *L* = 1.4*m*, *k*_*ds*_ = 500*N*/*m*, *k*_*hs*_ = 10000*N*/*m*) and low stiffness condition *k*_1,2_ = 500*N*/*m* (in red, using the WSNR model and *L* = 1.4*m*, *k*_*ds*_ = 500*N*/*m*, *k*_*hs*_ = 10000*N*/*m*). Simulation results for: (A) the pole angle, (B) control force at the hand, and the interaction force at the shoulder in (C) forward direction and (D) vertical direction are shown.

We tested a low stiffness value (*k*_1,2_ = 500 *N*/*m*), which is believed to be beneficial during walking by reducing energetic cost and peak interaction force [15]. We compared results with the no-spring condition (Fig 10). The pole angle *θ* and the control force show significantly smaller amplitude at a low stiffness value, compared to the no-spring condition. The interaction force (on the shoulder) in the vertical direction shows a much smaller amplitude at low stiffness, with little change in the forward direction.

**Fig 11:**
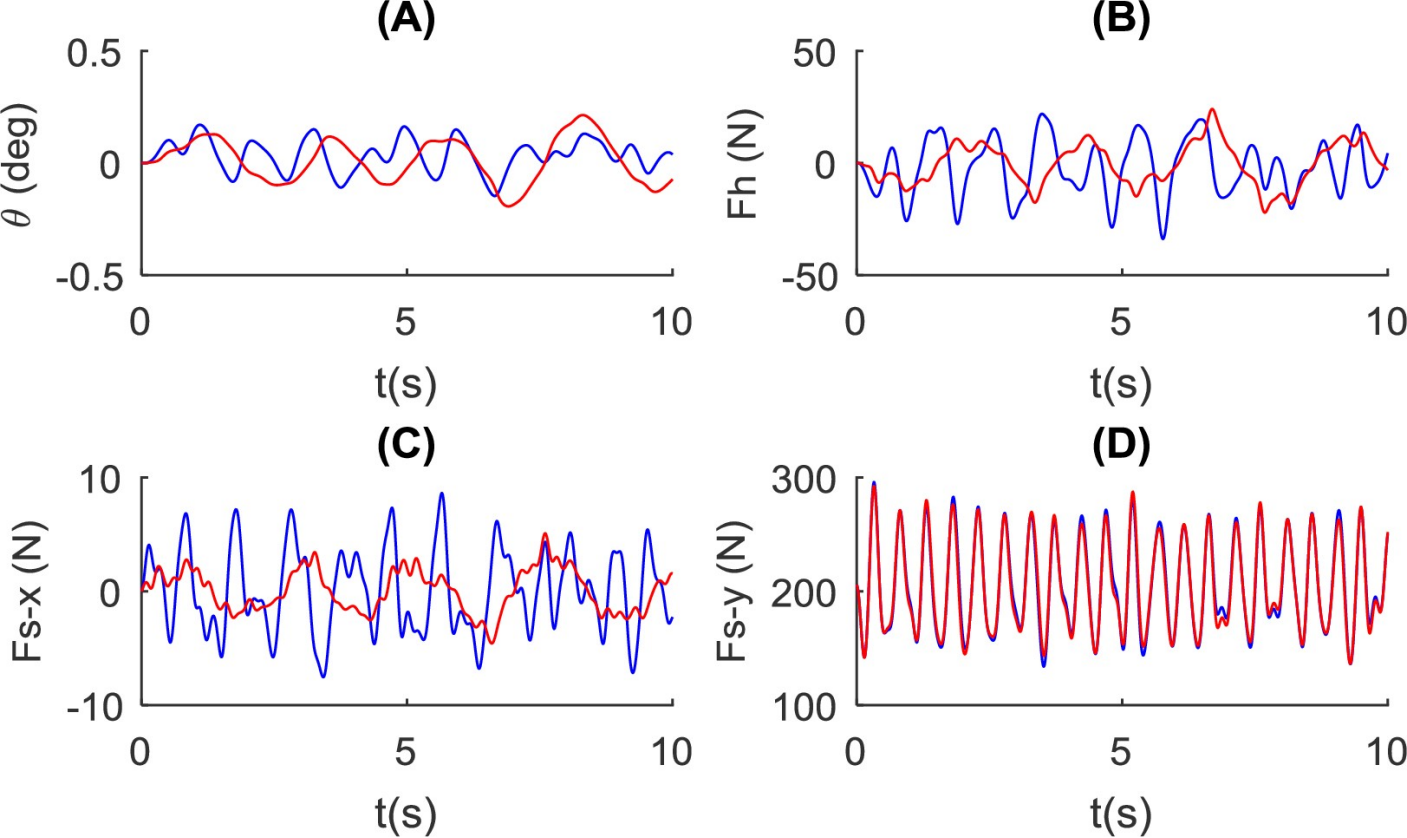
Influence of rope on the balance of the pole and pole-carrier interaction. The no rope condition (in blue, using the NSNR model and *L* = 1.4*m*, *k*_*ds*_ = 500*N*/*m*, *k*_*hs*_ = 10000*N*/*m*) and the condition with a rope length of 1m (in red, using the NSWR model and *L* = 1.4*m*, *k*_*ds*_ = 500*N*/*m*, *k*_*hs*_ = 10000*N*/*m*) are shown. Simulation results for: (A) the pole angle, (B) control force at the hand, and the interaction force at the shoulder in (C) forward direction and (D) vertical direction are shown.

The model is simulated with a rope length of 1.0 m for comparison with the no rope condition (Fig 11). The pole angle and the control force have much longer oscillation periods, but with similar amplitude, compared to the no rope condition. The fore-aft interaction force is also different for the no rope condition, showing a much longer period and smaller amplitude. However, the vertical interaction forces under the two conditions are almost identical.

**Fig 12:**
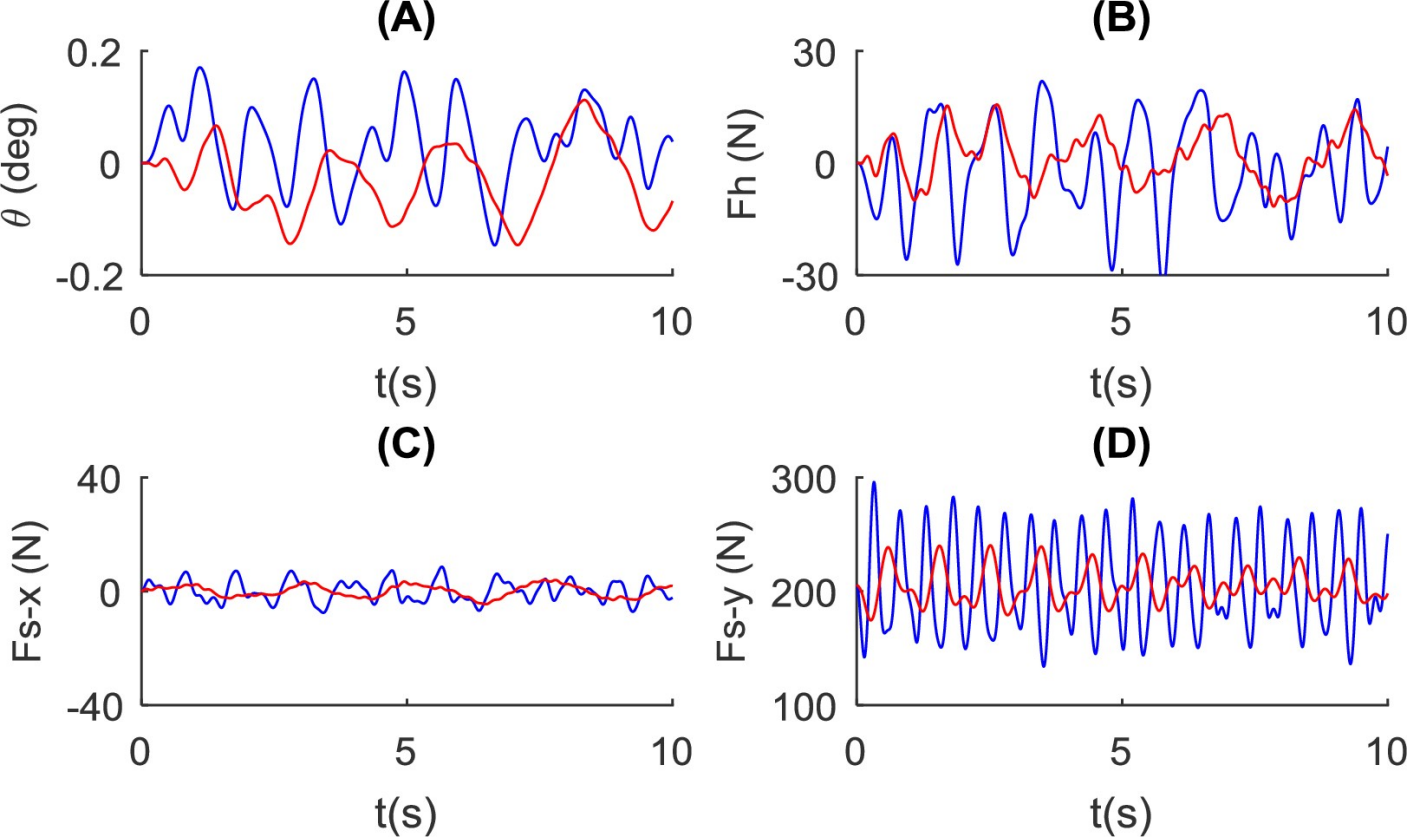
The combined influence of pole stiffness and rope on pole balance and pole-carrier interaction. The comparison is made between the condition of no spring and no rope (in blue, using the NSNR model and *L* = 1.4*m*, *k*_*ds*_ = 500*N*/*m*, *k*_*hs*_ = 10000*N*/*m*) and the condition of pole stiffness *k*_1,2_ = 500 *N*/*m* and rope length *L*_*r*1,2_ = 1*m* (in red, using the WSWR model and *L* = 1.4*m*, *k*_*ds*_ = 500*N*/*m*, *k*_*hs*_ = 10000*N*/*m*). Simulation results for: (A) the pole angle, (B) control force at the hand, and the interaction force at the shoulder in (C) forward direction and (D) vertical direction are shown.

**Fig 13:**
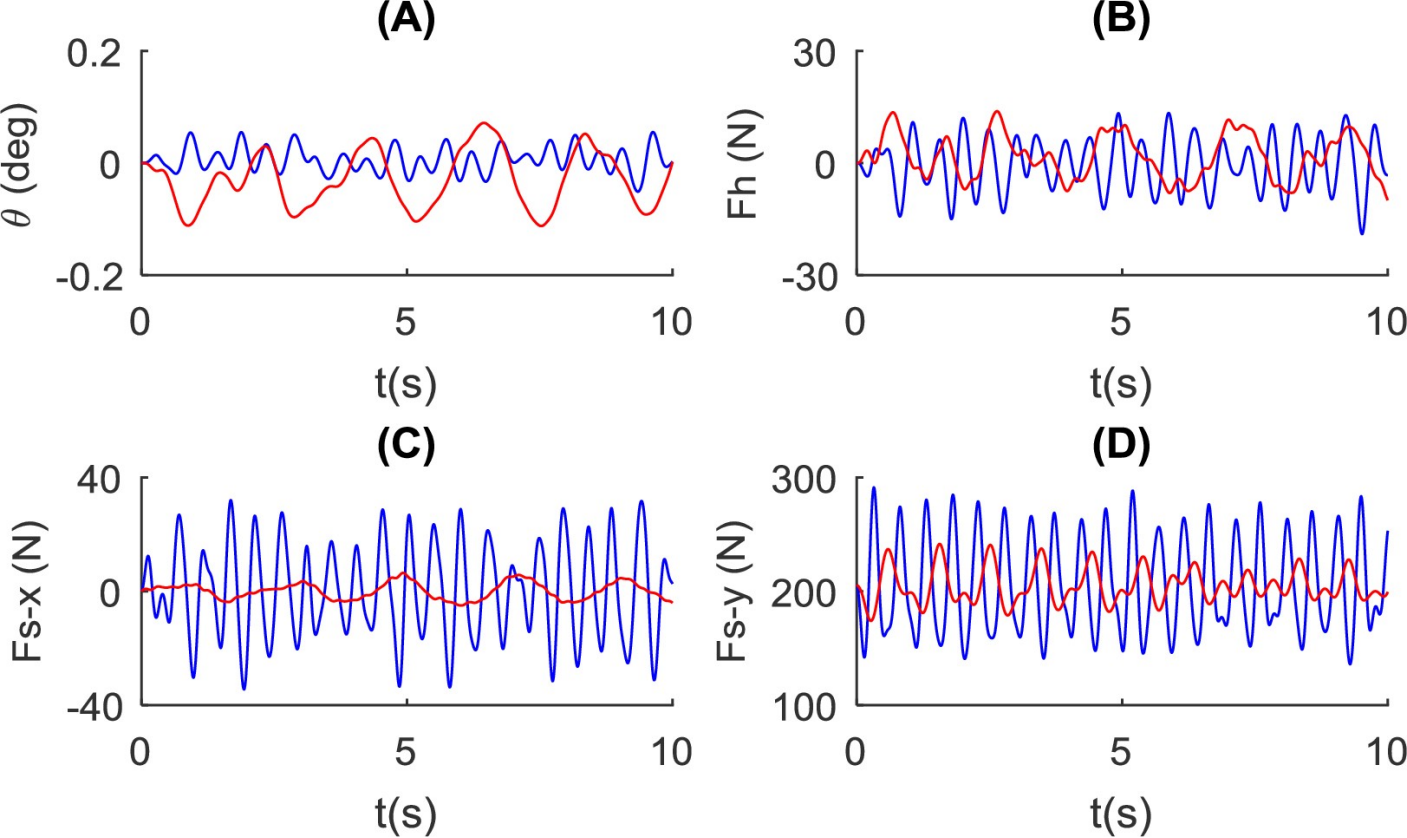
The combined influence of pole stiffness and rope on pole balance and pole-carrier interaction. The comparison is made between the condition of no spring and no rope (in blue, using the NSNR model and *L* = 1.4*m*, *k*_*ds*_ = 4000*N*/*m*, *k*_*hs*_ = 10000*N*/*m*) and the condition of pole stiffness *k*_1,2_ = 500 *N*/*m* and rope length *L*_*r*1,2_ = 1*m* (in red, using the NSNR model and *L* = 1.4*m*, *k*_*ds*_ = 4000*N*/*m*, *k*_*hs*_ = 10000*N*/*m*). Simulation results for: (A) the pole angle, (B) control force at the hand, and the interaction force at the shoulder in (C) forward direction and (D) vertical direction are shown.

The model exhibiting low stiffness (500N/m) and a long rope (1m) shows combined effects of the above two conditions (Fig 12). The pole angle and control force variation have a longer period. The fore-aft interaction has a longer period and a smaller amplitude. The vertical interaction force also shows significantly reduced amplitude. The WSWR model is compared with the NSNR model at a different contact stiffness *k*_*ds*_ (4000N/m) in Fig 13. The interaction force and pole angle show a similar trend, although the pole angle in the NSNR model is significantly smaller than that using *k*_*ds*_ = 500*N*/*m*.

## Discussion

The carrying pole’s long history of application has been documented in many different places around the world. It was used for a relatively short period of time in many places but continues to be popular in some underdeveloped areas in Asia. We believe the prevalence of the carrying pole in East Asia is a blended consequence of many aspects including history, economics, engineering, and environment. However, it is not the focus of this paper to explain the prevalence of the carrying pole, as it requires knowledge across multiple disciplines. Our goal is to explore the mechanics of the carrying pole to reveal the operational principles of the carrying pole and the influence of its structure and parameters on pole balance and human-pole interaction. Full understanding of its mechanism may also help to explain why people would prefer this tool over some other load carriage tools given certain conditions, which accounts as an essential step to explain the popularity of the carrying pole.

### Human-pole Interface

The shoulder and hand interfaces play a crucial role in load carriage with a pole. Analyzing the influence of pole properties on gait, and the body, requires the knowledge of inputs and control at the human-pole interface. With little available knowledge, we simplified the interaction at the shoulder and hand by choosing a spring-damping mechanism and PD controller to represent these mechanisms. The exact parameters of the contact model are indeed unknown and thus we examined the simulation with a range of parameters. We showed the influence of pole parameters on pole tipping and pole-human interface exhibit a similar trend (Fig 13). Different models for representing contact (such as friction models) may also be examined to better explain the pole-shoulder contact in future work. Carrying the pole also requires the complex coordination of the shoulder and hand, which are still unclear. Although the simple PD controller may not capture the real control strategy, it still helps to show that pole parameters will have a large impact on pole balance control.

### Pole length

The length of the pole was shown to affect pole carrying dynamics as it alters the rotational inertia of the pole-load system. Longer poles exhibit slower roll-over speed during simulations with either step input or measured shoulder trajectory (Figs 6B, 8A and 9A). As well, the control force also tends to be smaller for longer poles (Fig 9B). This could be beneficial for load carriage as it allows the carrier to respond slowly or with a delay. However, the effect size of pole length in the dynamic condition is rather small, indicating the choice of length may depend on practical factors and load dimension.

### Pole stiffness

Previous studies have considered the stiffness to be a major property of the carrying pole [5, 14–16]. It is believed that the stiffness should be very low and that the natural frequency of the pole-load system should be lower than the walking frequency [14, 15]. The major benefit of a low stiffness pole is that it the carried load oscillates out of phase with the body, which reduces peak interaction force in the vertical direction as also observed in our simulation (Fig 10). The out-of-phase motion is also believed to be the source of reduced energetic cost when walking with a springy pole [14, 17]. We believe stiffness could influence the energetics of gait to some extent, but it is more likely to be constrained by the trade-off in pole dimension and weight. Although a lower pole stiffness can reduce the vertical interaction force amplitude, and therefore reduce the impact on the shoulder, it may require a different control technique. We observed that the frequency of the control force tends to be higher in conditions with lower pole stiffness (Fig 10). This indicates that the stiffness will change the balance control strategy and requires further exploration.

### Rope length

The function of the rope is discussed in the previous section from an ergonomic perspective. In the step response test (Fig 8C), the rope leads to a longer settling time, especially at shorter rope conditions. This is probably because the rope swing is energy-conservative with little damping effect, which is much harder to dissipate within a small number of oscillation cycles. A longer rope causes a longer oscillation period which explains the low-frequency oscillation of pole angle in Fig 11. It is also noted that a long rope reduces the interaction force in the fore-aft direction, which may reduce the induced torque on the lumbar joint. When combined with a vertical spring, the fore-aft and vertical interaction force at the shoulder can both be reduced (Fig 12C and 12D). However, our analysis indicates that the rope may pose challenges for control, especially with unsteady body motion input. This may also account for the observation that sometimes the carrier will hold the rope to achieve stable walking in rough terrain, especially when carrying a liquid load such as water.

## Conclusion

In this paper, we investigated the mechanics of the carrying pole, a popular load-carrying tool in many Asian countries. We did not directly analyze the influence of pole carriage on gait energetics, as it requires a full understanding of the mechanics of the carrying pole, gait mechanics and human motor control. Instead, as the first step, we focused on understanding the mechanics of the carrying pole that could potentially affect load carriage performance. Through simulation, we showed that this seemingly simple tool produces rather complex behavior. Different from other load-carrying tools like backpacks, the carrying pole requires the carrier to keep the pole’s balance which is essential during walking. We analyzed multiple factors including pole length, pole stiffness, and rope length. We found that these factors have large influences on pole balance and the human-pole interaction. The current study is still limited by the little knowledge of the pole-shoulder contact and human motor control strategy. Even so, it is believed that these structural and dynamic analyses can serve as a foundation for further development of human-pole interaction models. We hope this study can inspire more studies of this load carrying tool and point to potential directions for further investigation.

## Appendix A

The EOM of the system is obtained via the Newton-Euler Method.

According to the model diagram in Fig 4, the body motion is given as **p**_**o**_ = [*x*_*o*_ *y*_*o*_]^*T*^, the center of mass of the pole is **p**_**p**_ = **p**_**o**_ + [−*d*_*s*_ cos *θ h*_*s*_−*d*_*s*_ sin *θ*]^*T*^. The position of the two loads are **p**_**1**_ = **p**_**p**_ + [−*L*_*p*1_ cos *θ* + *L*_*r*1_ sin *α*_1_ −*L*_*p*1_ sin *θ* −*L*_*r*1_ cos *α*_1_−*h*_1_]^*T*^,**p**_**2**_ = **p**_**p**_ + [*L*_*p*2_ cos *θ* + *L*_*r*2_ sin *α*_2_ *L*_*p*2_ sin *θ* −*L*_*r*2_ cos *α*_2_−*h*_2_]^*T*^ and pole-carrier interaction points are given as **p**_**s**_ = **p**_**o**_ + [0 *h*_*s*_]^*T*^,**p**_**h**_ = **p**_**p**_ + [*D*_*h*_ cos *θ D*_*h*_ sin *θ*]^*T*^.

From the linear momentum balance:

$$F_{1}+m_{1}{\left[0\;-g\right]}^{T}=m_{1}\ddot{p_{1}}$$ (a1)

$$F_{2}+m_{2}{\left[0\;-g\right]}^{T}=m_{2}\ddot{p_{2}}$$ (a2)

$$F_{s}-F_{1}-F_{2}+{\left[0\;F_{h}\right]}^{T}=m_{0}\ddot{p_{p}}$$ (a3)

From the angular momentum balance:

$$F_{1-y}(p_{1-x}-p_{p-x})-F_{1-x}(p_{1-y}-p_{p-y})+F_{2-y}(p_{2-x}-p_{p-x})-F_{2-x}(p_{2-y}-p_{p-y})+F_{s-y}(p_{s-x}-p_{p-x})-F_{s-x}(p_{s-y}-p_{p-y})+F_{h}(p_{h-x}-p_{p-x})=I_{0}\ddot{\theta }$$ (a4)

where the subscripts ‘-x’ and ‘-y’ denote the x and y components of the vector.

From the spring and rope mechanism, we also have:

$$F_{s}={\left[\frac{k_{ds}d_{s}+c_{ds}\dot{d_{s}}+(k_{hs}h_{s}+c_{hs}\dot{h_{s}})\sin\theta }{\cos\theta }\;-(k_{hs}h_{s}+c_{hs}\dot{h_{s}})\right]}^{T}$$ (a5)

$$F_{1}={\left[-(k_{1}h_{1}+c_{1}\dot{h_{1}})\tan\alpha_{1}\;k_{1}h_{1}+c_{1}\dot{h_{1}}\right]}^{T}$$ (a6)

$$F_{2}={\left[-(k_{2}h_{2}+c_{2}\dot{h_{2}})\tan\alpha_{2}\;k_{2}h_{2}+c_{2}\dot{h_{2}}\right]}^{T}$$ (a7)

Plug (a5)~(a7) into (a1)~(a3) and we can have 6 equations. Combining (a4), we have seven equations (a8) with seven general coordinates **x** = (*h*_*s*_,*d*_*s*_,*θ*,*h*_1_,*θ*_1_,*h*_2_,*θ*_2_).

$$M(x)\ddot{x}+C(x,\dot{x})+G(x)=B_{1}\left[\begin{array}{c} \ddot{x_{o}}\\ \ddot{y_{o}} \end{array}\right]+B_{2}[F_{h}]$$ (a8)

## Supporting information

S1 File The experimentally measured shoulder motion data used in simulations. (XLSX)
